# 免疫治疗在EGFR突变晚期非小细胞肺癌中的研究进展

**DOI:** 10.3779/j.issn.1009-3419.2023.106.26

**Published:** 2023-12-20

**Authors:** Yaoyao LIU, Jianlong MIAO

**Affiliations:** ^1^272000 济宁，济宁医学院临床医学院（刘尧尧）; ^1^Clinical Medical College of Jining Medical University; ^2^济宁市第一人民医院呼吸与危重症医学科（苗健龙）; ^2^Pulmonary and Critical Care Medicine, Jining No. 1 People’s Hospital, Jining 272000, China

**Keywords:** 肺肿瘤, 表皮生长因子受体基因突变, 免疫治疗, Lung neoplasms, Epidermal growth factor receptor (EGFR) gene mutation, Immunotherapy

## Abstract

表皮生长因子受体（epidermal growth factor receptor, EGFR）基因突变晚期非小细胞肺癌（non-small cell lung cancer, NSCLC）的一线治疗方案首选EGFR酪氨酸激酶抑制剂（tyrosine kinase inhibitors, TKIs），但耐药不可避免。近几年，虽然免疫检查点抑制剂（immune checkpoint inhibitors, ICIs）改变了无驱动基因突变晚期NSCLC的治疗模式，但这些药物对EGFR突变晚期NSCLC患者的临床疗效有限。与EGFR野生型肿瘤相比，EGFR突变型肿瘤在程序性细胞死亡配体1（programmed cell death ligand 1, PD-L1）、肿瘤突变负荷（tumor mutational burden, TMB）等肿瘤微环境（tumor microenvironment, TME）的特征上更具异质性，ICIs是否适用于EGFR突变晚期NSCLC患者仍值得探讨。这篇综述总结了有关ICIs在EGFR突变晚期NSCLC患者中疗效的临床数据，并解读了EGFR突变NSCLC独特的TME。

## Abstract (en)

Epidermal growth factor receptor-tyrosine kinase inhibitors (EGFR-TKIs) are currently the first-line standard of care for patients with non-small cell lung cancer (NSCLC) that harbor EGFR mutations. Nevertheless, resistance to EGFR-TKIs is inevitable. In recent years, although immune checkpoint inhibitors (ICIs) have significantly shifted the treatment paradigm in advanced NSCLC without driver mutation, clinical benefits of these agents are limited in patients with EGFR-mutated NSCLC. Compared with wild-type tumors, tumors with EGFR mutations show more heterogeneity in the expression level of programmed cell death ligand 1 (PD-L1), tumor mutational burden (TMB), and other tumor microenvironment (TME) characteristics. Whether ICIs are suitable for NSCLC patients with EGFR mutations is still worth exploring. In this review, we summarized the clinical data with regard to the efficacy of ICIs in patients with EGFR-mutated NSCLC and deciphered the unique TME in EGFR-mutated NSCLC.

表皮生长因子受体（epidermal growth factor receptor, EGFR）基因经典突变（19号外显子缺失或L858R点突变）于2004年被确定为非小细胞肺癌（non-small cell lung cancer, NSCLC）中首个可靶向治疗的致癌驱动基因^[1]^。靶向药物酪氨酸激酶抑制剂（tyrosine kinase inhibitors, TKIs）的使用，改变了驱动基因阳性NSCLC的治疗模式，使患者获得了更高的应答率和良好的耐受性。根据美国国立综合癌症网络（National Comprehensive Cancer Network, NCCN）指南，EGFR-TKIs被推荐为EGFR突变晚期NSCLC患者的标准一线治疗药物。与标准化疗相比，第一代、第二代EGFR-TKIs的中位无进展生存期（median progression-free survival, mPFS）更长（9-13个月），反应率更高，副作用更轻，但并未显示出总生存期（overall survival, OS）方面的优势。对于第一代和第二代EGFR-TKIs治疗耐药后的T790M阳性晚期NSCLC患者，与铂类化疗相比，第三代EGFR-TKIs（奥希替尼）显示出更高的疗效和更低的严重不良反应发生率，且mPFS为18.9个月^[2⇓⇓-5]^。虽然靶向治疗显著提高了患者的生存率，但对大多数患者来说，长期应答仍不常见，EGFR-TKIs的耐药性不可避免^[6]^。在过去的几年中，免疫检查点抑制剂（immune checkpoint inhibitors, ICIs）极大地提高了无驱动基因突变NSCLC患者的生存率^[7,8]^，但免疫单药对EGFR突变的NSCLC患者并没有明显改善疗效^[9]^。与EGFR野生型肿瘤相比，EGFR突变型肿瘤在程序性细胞死亡配体1（programmed cell death ligand 1, PD-L1）、肿瘤突变负荷（tumor mutational burden, TMB）等肿瘤微环境（tumor microenvironment, TME）特征上更具异质性^[10]^。ICIs是否适用于EGFR突变的NSCLC患者仍值得探讨。因此，本文旨在对免疫治疗在EGFR突变晚期NSCLC的疗效进行综述，以期指导临床治疗。

## 1 免疫治疗在EGFR突变晚期NSCLC中的临床研究现状

### 1.1 免疫单药治疗

在KEYNOTE-001 I期试验中，接受帕博利珠单抗（Pembrolizumab）治疗的初治EGFR突变患者（n=4）的mPFS（157.5 vs 56 d）和中位OS（median OS, mOS）（559 vs 120 d）均长于此前接受EGFR-TKIs治疗的患者（n=26），然而KEYNOTE-001的II期试验^[11]^却没有达到预期的类似结果，共招募了25例EGFR突变的初治NSCLC患者，其中11例接受了Pembrolizumab治疗，但该试验因缺乏疗效[客观缓解率（objective response rate, ORR）为4%，mPFS为56 d，mOS为12 d]而终止。CheckMate017/057、KEYNOTE-010、OAK和POPLAR等研究^[12⇓⇓-15]^显示，在二线或后续治疗中，免疫疗法似乎对EGFR突变的NSCLC患者无效。两项荟萃分析中发现，与多西他赛相比，在EGFR野生型亚组中，纳武利尤单抗（Nivolumab）、Pembrolizumab和阿替利珠单抗（Atezolizumab）与OS延长相关，但在EGFR突变亚组中却不相关^[9,16]^。CheckMate012试验^[17]^共纳入52例患者，在亚组分析中显示与EGFR野生型患者（n=30）相比，Nivolumab对EGFR突变患者（n=7）的疗效较差（ORR：30% vs 14%；mPFS：8.8 vs 1.8个月；mOS：未达到 vs 18.8个月）。ATLANTIC^[18]^是一项II期、开放、单臂临床试验，将度伐利尤单抗（Durvalumab）作为晚期NSCLC的三线或更晚期治疗，共有444例患者入组，亚组分析显示虽然PD-L1表达≥25%的EGFR突变型NSCLC的ORR高于低表达组[12.2% (9/74)_PD-L1≥25%_ vs 3.6% (1/28)_PD-L1<25%_]，但仍比EGFR野生型组群差[16.4% (24/146)_PD-L1≥25%_ vs 7.5% (7/93)_PD-L1<25%_]。BIRCH研究^[19]^旨在评估Atezolizumab在晚期NSCLC患者中的疗效，研究亚组共纳入了45例EGFR突变患者，尽管结果显示Atezolizumab对EGFR突变型和EGFR野生型肿瘤均有抗肿瘤活性，但与野生型肿瘤相比，EGFR突变型的有效性较低。总之，这些临床试验的数据提示，对于EGFR突变的NSCLC患者一线或后线单药免疫治疗在PD-L1高表达的人群中可能会获益，但仍比EGFR野生型疗效差。

### 1.2 免疫双药治疗

抗程序性细胞死亡受体1（programmed cell death protein 1, PD-1）抗体和抗细胞毒T淋巴细胞相关蛋白4（cytotoxic T-lymphocyte-associated protein 4, CTLA-4）抗体以不同但互补的方式调节抗肿瘤免疫反应。美国食品和药物监督管理局已批准将Nivolumab联合伊匹单抗（Ipilimumab）和2个周期的铂类双药化疗作为一线治疗无EGFR或间变性淋巴瘤激酶（anaplastic lymphoma kinase, ALK）基因突变的转移或复发性NSCLC患者^[20]^。CheckMate012研究^[21]^共纳入了78例患者，其中一项亚组分析了Nivolumab联合Ipilimumab在EGFR突变组（n=8）中的效果，其中50%的患者获得了客观应答，高于EGFR野生型患者（n=54）的ORR（41%）。在KEYNOTE021研究^[22]^（n=51）的一个亚组中，当接受Ipilimumab和Pembrolizumab联合治疗时，EGFR-TKIs耐药后的EGFR突变型NSCLC（n=10）的ORR仅为10%，而EGFR野生型组（n=33）为30%。以上试验表明，双ICIs在EGFR突变NSCLC中的疗效还需要进一步证实。

### 1.3 免疫联合靶向治疗

临床前研究^[23]^表明，与未接受厄洛替尼（Erlotinib）治疗的诱导EGFR突变肿瘤的小鼠相比，接受厄洛替尼治疗的小鼠肿瘤中浸润的调节性T细胞（regulatory T cells, Tregs）数量较少，而细胞毒性CD8^+ ^T细胞数量较多，这表明肿瘤组织的炎症程度更高。此外，与单独使用其中一种疗法相比，厄洛替尼和抗PD-1疗法联合使用可显著抑制EGFR突变小鼠肿瘤的生长。因此，联合使用EGFR靶向疗法有可能提高ICIs在EGFR突变NSCLC中的疗效。然而，为探索EGFR-TKIs和ICIs联合使用的安全性和疗效而开展的几项早期临床试验都因严重的肝脏或肺部毒性而提前终止。TATTON^[24]^和CAURAL^[25]^试验探索了奥希替尼和Durvalumab在EGFR突变NSCLC中的联合用药，两项研究均因间质性肺病的发病率较高而提前终止。在KEYNOTE-021研究^[26]^中，共纳入19例初治EGFR突变型NSCLC患者，其中12例患者接受了Pembrolizumab+厄洛替尼治疗，ORR和mPFS分别为41.7%和19.5个月，且不良事件（adverse events, AEs）与接受单药治疗的患者相似；但接受Pembrolizumab和吉非替尼联合疗法的组别显示出较高的肝毒性（3/4级）发生率（5/7, 71.4%）。一项临床研究（n=21）^[27]^评估了Nivolumab联合厄洛替尼作为二线疗法在EGFR突变患者中的疗效，AEs发生率没有明显增加，但临床疗效也没有改善。在一项I期临床试验^[28]^中，Ipilimumab联合厄洛替尼明显改善了EGFR突变晚期NSCLC患者（n=11）的mPFS（27.8个月）和mOS（未达到，但是>42.3个月），但因引起胃肠道毒性过大，导致研究提前结束。在一线和其他治疗方案中，都观察到了ICIs联合EGFR-TKIs的严重AEs。联合疗法并没有进一步提高疗效，反而给患者带来了更多的安全风险。因此，EGFR-TKIs和ICIs 联合治疗的机制、安全性和疗效有待进一步研究。

### 1.4 免疫联合化疗、抗血管生成治疗

#### 1.4.1 免疫+化疗

在无驱动基因突变的晚期NSCLC中，ICIs与化疗的联合治疗显示出比单独化疗更好的mPFS和mOS^[29]^。目前，联合方案已被列为无EGFR/ALK突变的晚期NSCLC的一线治疗方案。受此启发，有人尝试将免疫疗法与化疗相结合，用于治疗EGFR突变的晚期NSCLC患者。几项早期研究显示，ICIs+化疗对EGFR-TKIs治疗进展的NSCLC患者具有良好的抗肿瘤活性。一项II期临床研究^[30]^评估了Pembrolizumab联合卡铂和培美曲塞治疗复发性EGFR突变NSCLC（n=26）的疗效，结果显示ORR为42.3%，mPFS为8.3个月，mOS为22.2个月。同样，一项研究^[31]^评估了替雷利珠单抗（Tislelizumab）联合化疗对EGFR-TKIs治疗失败的EGFR突变晚期非鳞状NSCLC患者（n=32）的疗效，初步分析显示ORR和疾病控制率（disease control rate, DCR）分别为59.4%（95%CI: 40.6%-76.3%）和90.6%（95%CI: 75.0%-98.0%），约32.5%的患者出现了3级以上的相关AEs。CT18^[32]^是一项II期临床研究，评估了特瑞普利单抗（Toripalimab）与培美曲塞/卡铂联合治疗对EGFR-TKIs耐药且无T790M突变的EGFR突变患者（n=40）的效果：该方案的mPFS为7个月，mOS为23.5个月，ORR 为50%，DCR为87.5%，只有2例患者（5.0%）发生了≥3 级的虹膜不良反应，包括1例（2.5%）皮肤毒性和1例（2.5%）肺炎。Yang等^[33]^研究发现，对于EGFR-TKIs治疗失败后的EGFR突变晚期NSCLC患者，免疫+化疗联合组（n=25）的疗效优于免疫单药组（n=6）（mPFS: 3.42 vs 1.58个月，P=0.027），未观察到≥3级的免疫治疗相关AEs。综上，对于EGFR-TKIs耐药的EGFR突变NSCLC患者来说，在标准化疗的基础上增加免疫疗法有可能会提高疗效。

#### 1.4.2 免疫+抗血管生成治疗

血管内皮生长因子（vascular endothelial growth factor, VEGF）和EGFR是促进肿瘤进展和转移的关键因素，并具有共同的下游信号通路。EGFR信号通路可诱导VEGF的表达，从而调节肿瘤特异性血管生成，促进髓源性抑制细胞向肿瘤内迁移，从而促进免疫抑制TME的形成^[34⇓-36]^。抗VEGF抗体不仅能促进肿瘤血管正常化，还能解除免疫抑制性TME^[36]^。一项研究^[37]^表明，ICIs联合抗血管生成药物可通过促进CD8^+ ^T细胞在NSCLC中的浸润和细胞毒性而诱导持久的抗癌效果，研究表明，联合使用抗血管生成药物可能有助于扩大从免疫疗法中获益的优势人群。在一项II期队列研究^[38]^中，阿帕替尼联合卡瑞利珠单抗（Camrelizumab）治疗EGFR-TKIs 耐药后的EGFR突变NSCLC患者（n=43）显示出令人鼓舞的抗肿瘤活性和可接受的毒性（ORR：18.6%，95%CI：8.4%-33.4%；mPFS: 2.8个月，95%CI：1.9-5.5个月）。Wang等^[39]^在回顾性研究中发现，在复发性EGFR突变NSCLC患者（n=9）中，安罗替尼加抗PD-1治疗的mPFS为7.2个月，mOS为9.6个月，40%的患者发生了≥3级的治疗相关AEs，但没有致命AEs的报道。另一项回顾性研究^[40]^比较了Pembrolizumab单药治疗（PM组，n=32）、Pembrolizumab联合化疗（P+C组，n=26）和Pembrolizumab联合安罗替尼（P+A组，n=28）治疗EGFR-TKIs耐药后的EGFR突变NSCLC患者的疗效，与PM组相比，P+C组和P+A组分别在mPFS（分别为1.5、4.3和3.24个月）、mOS（分别为7.41、14.92和15.97个月）和ORR（分别为3.1%、23.1%和 21.4%）方面取得了更好的临床疗效。以上研究结果表明，免疫+抗血管生成治疗对EGFR-TKIs耐药的EGFR突变NSCLC患者具有潜在疗效。然而，由于样本量较小，且现有研究较少，免疫+血管生成疗法的联合应用值得进一步研究。

#### 1.4.3 免疫+化疗+抗血管生成治疗

在IMpower 150研究^[41]^中，124例EGFR-TKIs耐药的NSCLC亚组患者接受了ACP（Atezolizumab、卡铂和紫杉醇）、BCP（贝伐珠单抗、卡铂和紫杉醇）和ABCP（Atezolizumab、贝伐珠单抗、卡铂和紫杉醇）方案治疗。ACP、BCP和ABCP方案的mPFS分别为6.9、6.9和10.2个月，mOS分别为19.0、18.1和29.4个月。这些结果表明，ABCP联合方案可能是治疗EGFR-TKIs耐药晚期NSCLC患者的一种新疗法。此外，ABCP、ACP和BCP治疗组分别有66.7%、56.8%和55.8%的患者出现3/4级治疗相关AEs。ABCP、ACP和BCP组分别有42.4%、13.6%和16.3%的患者因AEs而放弃治疗。一项II期临床研究^[42]^纳入了40例EGFR-TKIs治疗后进展的NSCLC患者，他们接受了Atezolizumab、贝伐珠单抗、培美曲塞和卡铂的联合治疗方案，ORR为62.5%，mPFS为9.4个月，1年生存率为72.5%，与IMpower 150研究^[41]^的结果相似，37.5%的患者出现了3级或以上的治疗相关AEs，32.5%的患者出现了免疫相关AEs。ORIENT-31的III期研究^[43]^旨在探索信迪利单抗（Sintilimab）±IBI305联合化疗（培美曲塞+顺铂）对EGFR-TKIs治疗失败后的EGFR突变局部晚期或转移非鳞状NSCLC患者的疗效，中位随访时间为9.8个月，结果显示Sintilimab+IBI305+化疗组（n=148）比单纯化疗组（n=151）有更长的mPFS（6.9 vs 4.3个月，HR=0.464，95%CI: 0.337-0.639，P<0.0001），Sintilimab+化疗组（n=145）的mPFS为5.6个月。Sintilimab+IBI305+化疗组的3级或4级中性粒细胞减少发生率为20%，而Sintilimab+化疗组为18%，单纯化疗组为18%。以上研究表明，将免疫疗法、化疗和抗血管生成结合起来可能对EGFR-TKIs耐药的EGFR突变NSCLC患者是一种很有前景的治疗策略。

## 2 EGFR突变晚期NSCLC的免疫学特征

在亚洲人群中，晚期肺腺癌患者EGFR突变频率（51.4%）要显著高于白人（约20%），且在不吸烟（60.7%）的女性（61.1%）患者中更常见^[44]^。一项纳入了1200例中国NSCLC患者的研究^[45]^中发现，EGFR突变和ALK重排频率分别为50.1%和7.8%，显著高于西方人群。在18.9%的EGFR突变型NSCLC患者中发现了除19号外显子缺失、L858R点突变、20号外显子插入和T790M突变以外的56种不同的罕见EGFR突变，约7.4%的患者同时携带敏感突变和罕见突变，11.6%的患者仅携带罕见突变。EGFR罕见突变多合并ALK、CDKN2A、NTRK3、TSC2和KRAS突变。尽管ICIs已大大改变了晚期驱动基因阴性NSCLC的治疗模式，但这些药物对EGFR突变NSCLC患者的临床疗效有限。一些疗效预测性生物标志物（如PD-L1、TMB等）已在EGFR突变野生型NSCLC中得到验证，但在EGFR突变型肿瘤中却没发现相似的作用，这表明EGFR突变型NSCLC的TME更具独特性。

### 2.1 EGFR突变NSCLC的PD-L1表达情况

#### 2.1.1 EGFR突变和PD-L1表达

目前EGFR突变与PD-L1表达的调控仍存在争议。Azuma等^[46]^首次报道了在手术切除的NSCLC中，PD-L1在EGFR突变肿瘤中的表达显著高于EGFR野生型肿瘤（P<0.001）。同时也有实验数据^[47]^表明EGFR突变可能通过5种途径（如Ras/RAF/MEK/ERK、PI3K/AKT/mTOR、JAK/STAT、NF-κB和YAP）上调PD-L1的表达。而专门针对EGFR突变的一项对15个临床试验的汇总分析^[48]^中发现，EGFR野生型肿瘤比EGFR突变型更容易检测到PD-L1的表达（P=0.02），他们进一步评估了癌症基因组图谱（The Cancer Genome Atlas, TCGA）和内部数据库（广东省肺癌研究所-GLCI）中切除的NSCLC组织中PD-L1的mRNA和蛋白水平，同样发现EGFR突变肿瘤中PD-L1的表达低于EGFR野生型肿瘤[P_（TCGA）_=0.014; P _(GLCI）_=0.044]。Schoenfeld^[49]^和Lamberti^[50]^分别对1586例肺腺癌患者和909例非鳞状细胞癌患者纳入研究，得出了相同的结论：EGFR野生型肿瘤中PD-L1的表达率明显高于EGFR突变型肿瘤[P_（Schoenfeld）_<0.001; P_（Lamberti）_<0.001]。这一现象可能是由于EGFR通路信号持续活跃导致IRF1失活，从而抑制了γ-干扰素（interferon-gamma, IFN-γ）诱导的PD-L1表达^[23]^。以上结果不一致可能与多种因素有关，如不同的PD-L1检测技术、肿瘤异质性和患者肿瘤组织来源等。因此，PD-L1表达与EGFR突变的关联性尚不明确，仍需进一步研究。

#### 2.1.2 EGFR-TKIs导致PD-L1表达的变化

Jia等^[51]^在诱导的EGFR突变肺癌小鼠模型试验中发现，EGFR-TKIs可通过抑制EGFR信号转导来下调PD-L1的表达。然而，一些临床分析^[52⇓-54]^表明，EGFR-TKIs治疗后PD-L1的表达呈上升趋势。一项回顾性研究^[53]^分析了138例EGFR突变NSCLC患者的PD-L1表达情况，这些患者在EGFR-TKIs治疗进展后再次接受活检，发现PD-L1表达水平≥50%的患者比例从EGFR-TKIs治疗前的14%增加至28%（P=0.001）。其中27例患者EGFR-TKIs治疗进展后接受ICIs治疗，发现PD-L1高表达患者的OS长于PD-L1低表达患者（7.1 vs 1.7个月，P=0.003）。Gainor等^[54]^检测了EGFR-TKIs治疗前和EGFR-TKIs耐药后肿瘤组织中的PD-L1水平，同样证实，21%的患者（n=12）在EGFR-TKIs治疗耐药后，其肿瘤组织中的PD-L1表达增加。肿瘤细胞中PD-L1表达增加可能是EGFR-TKIs耐药患者二线免疫疗法取得较好疗效的部分原因。

### 2.2 EGFR突变NSCLC的TMB

TMB是指肿瘤基因编码区每兆碱基的体细胞上置换、插入和缺失突变的总数，它是预测免疫疗法疗效的良好生物标志物，可定量估计肿瘤基因组编码区的突变总数。TMB越高，肿瘤产生的新抗原越多，免疫细胞越容易识别，临床反应越持久^[55]^。肿瘤新抗原是通过主要组织相容性复合体分子呈现的方式，被T细胞受体（T-cell receptor, TCR）识别，从而诱导T细胞启动抗肿瘤反应程序^[56]^。在以往研究^[57]^的基础上，较高的TMB可以通过增加新抗原的数量来增强肿瘤的免疫原性，并与免疫治疗的疗效相关联。Hastings等^[58]^收集了来自耶鲁癌症中心（Yale Cancer Center, YCC）、纪念斯隆-凯特林癌症中心（Memorial Sloan Kettering Cancer Center, MSKCC）和TCGA的383例EGFR突变肺癌患者的测序数据，发现与EGFR野生型患者相比，EGFR突变患者的TMB水平较低，EGFR突变患者的TMB中位数为3.8个非同义突变/Mb，远低于野生型患者7.4个非同义突变/Mb。Dong等^[48]^分析了TCGA的TMB数据，同样显示EGFR突变组的TMB相对低于EGFR野生型组（TMB中位数: 56 vs 181，P<0.001），在另外两个数据库（Broad和GLCI）中，EGFR突变组的TMB也较低（Broad: 59 vs 209, P=0.003; GLCI: 162 vs 197, P=0.029）。在EGFR野生型NSCLC中，TMB水平低预示ICIs的疗效不佳^[59]^。TMB低可能是EGFR突变肿瘤中ICIs疗效不佳的一个潜在原因。

### 2.3 EGFR突变NSCLC的TME

TME是肿瘤生长和发展的内部环境，对免疫调节活动至关重要。EGFR突变NSCLC的TME通常具有低水平的CD8^+ ^T细胞和自然杀伤（natural killer, NK）细胞浸润、有限的TCR谱系以及大量的Tregs浸润，Tregs被认为是抗肿瘤免疫的一个关键障碍，肿瘤中Tregs分泌的转化生长因子-β（transforming growth factor beta, TGF-β）、白细胞介素（interleukin, IL）-10和IL-35可产生免疫抑制环境，主动削弱CD4^+ ^T细胞、CD8^+ ^T细胞和NK细胞的抗肿瘤免疫反应^[23,48,54,60]^。Mascia等^[61]^发现，敲除EGFR可显著抑制肿瘤细胞的生长，并下调Tregs在TME中的浸润。随着肿瘤的发展和免疫细胞的可塑性，T淋巴细胞通过免疫编辑从免疫监视功能转变为免疫逃逸功能，甚至表现出免疫抑制功能，如诱导Tregs和髓系抑制性细胞（myeloid-derived suppressor cells, MDSCs）^[62]^。此外，TME中的炎症细胞和免疫调节介质可能参与了介导肿瘤进展的重要机制，研究^[63]^发现EGFR突变型肿瘤细胞中富集的细胞因子[包括C-C趋化因子配体（chemokine CC-motif ligand, CCL）18、CXC趋化因子配体（C-X-C motif chemokine ligand, CXCL）1、CXCL3和IL-1β]大多具有招募和支持MDSCs、Tregs和肿瘤相关巨噬细胞（tumor-associated macrophages, TAMs）等免疫抑制活性细胞的功能，Tregs和TAMs同样表达高水平的免疫抑制细胞因子，而可募集效应T细胞的CCL3、CXCL9和CXCL10因子的水平则有所降低。与此相反，EGFR野生型肿瘤细胞则大量表达促炎细胞因子CXCL5、IL-7、IL-18和IL-32。CD73-腺苷信号通路被认为是导致TME免疫抑制的另一个重要机制，该通路可将AMP转化为腺苷，由此导致的细胞外腺苷过度累积会抑制CD8^+ ^T细胞、NK细胞、巨噬细胞和树突状细胞（dendritic cells, DCs）的抗肿瘤活性，同时促进Tregs和MDSCs的生成。与EGFR野生型NSCLC相比，EGFR突变型NSCLC表达更高水平的CD73 ^[64]^。

EGFR-TKIs可以缓解EGFR突变对T细胞的抑制，削弱Tregs的功能，促进IFN-γ的产生，并增强主要组织相容性复合体I分子（major histocompatibility complex class I, MHC-I）和MHC-II的表达^[65]^。然而，EGFR-TKIs对TME的影响可能是动态的，在小鼠模型中，Jia等^[51]^观察到EGFR-TKIs对TME的影响在早期是有益的，而在晚期则是免疫抑制，在早期阶段，CD8^+ ^T细胞、DCs和M1样TAMs的数量呈上升趋势，而Tregs浸润则下降；在EGFR-TKIs治疗后期，IL-10和CCL2分泌增加，促进了MDSCs 的迁移和活化，从而抑制免疫、促进血管生成和转移。Dominguez等^[66]^发现短期低剂量的厄洛替尼应用于EGFR突变肿瘤可产生免疫介导的细胞毒性以及基于NK细胞和抗原特异性T细胞的肿瘤裂解作用；然而，长期使用厄洛替尼治疗后，这种增强的免疫介导细胞毒性会消失。上述研究为在EGFR-TKIs耐药前使用ICIs治疗提供了依据。

综上，PD-L1的低表达、TMB的低水平以及免疫抑制TME的上调可能是EGFR突变NSCLC患者在接受ICIs治疗时处于劣势的原因。EGFR-TKIs对TME的影响可能是动态的，及时监测动态变化并选择适当的时机窗口可扩大免疫疗法的适用人群。靶向TME中的免疫调节因子也能提高免疫疗法的疗效。

## 3 EGFR突变晚期NSCLC免疫治疗疗效标志物

### 3.1 EGFR突变位点

#### 3.1.1 EGFR罕见突变

目前几项研究表明，EGFR罕见突变的NSCLC患者对ICIs有优先反应。一项纳入27例患者的回顾性研究^[67]^发现，与EGFR经典突变（19del/L858R）患者（n=20）相比，不常见EGFR突变（如G719X和20号外显子插入）患者（n=7）可从ICIs中更多获益，不常见亚型患者的ORR、DCR和mPFS更高（ORR: 71% vs 35.7%, P=0.14; DCR: 57% vs 7%, P<0.01; mPFS: 256 vs 50 d, P=0.003）。Chen等^[68]^的研究表明，NSCLC中EGFR罕见突变（包括20号外显子插入、G719X、L861Q或S768I）患者对ICIs的良好反应与其TME中PD-L1表达水平高和CD8^+ ^T浸润丰富有关。然而，目前针对每种突变类型的临床和临床前证据并不充分。尤其是对罕见突变的生物学特性了解不足。例如，EGFR 20号外显子插入突变具有多样性，而且往往与共突变相关^[69]^。这表明EGFR等位基因在ICIs治疗中的潜在差异，还需要进一步的前瞻性临床研究来验证。

#### 3.1.2 EGFR L858R突变

一项回顾性研究^[58]^报道，EGFR L858R突变患者（n=46）与EGFR野生型患者（n=212）对ICIs有相似的ORR（22% vs 16%, P=0.42）和OS（HR=0.917, 95%CI: 0.597-1.409, P=0.69）。然而，EGFR 19del患者（n=80）获益明显低于L858R突变患者（ORR: 7% vs 22%; OS: HR=0.69, P=0.03）。ICIs的疗效因PD-L1表达水平、TMB和T细胞浸润水平而异，Toki等^[70]^发现EGFR L858R突变患者TME中的活化T细胞数量高于EGFR 19del突变患者，并且L858R突变患者肿瘤中往往有较高的PD-L1表达和较高的TMB。

#### 3.1.3 T790M阴性

值得注意的是，与T790M阳性患者相比，T790M阴性NSCLC患者在EGFR-TKIs治疗期间疾病进展后接受Nivolumab治疗，具有更高的ORR和更长的PFS。这可能是因为与T790M阳性肿瘤相比，T790M阴性肿瘤具有更高的PD-L1表达水平、更高的CD8^+^T细胞数量和更低的Tregs^[71]^。Ma等^[72]^的研究中纳入了27例EGFR-TKI治疗耐药后的晚期NSCLC患者，接受了PD-1抑制剂加化疗和抗血管生成药物治疗，与T790M突变的患者（n=8）相比，T790M野生型的患者（n=19）的mPFS和ORR明显更好（9.2 vs 3.3个月，P=0.041；52.63% vs 12.5%，P=0.045）。此外，一项回顾性研究^[67]^显示，T790M阴性是EGFR突变的NSCLC患者使用ICIs有效率的积极预后因素。没有T790M突变的晚期NSCLC患者可能是标准EGFR-TKIs治疗失败后可从ICIs中获益的亚组。

### 3.2 PD-L1表达水平

研究^[73]^发现在ICIs治疗EGFR突变NSCLC队列（n=125）中，PD-L1阳性表达与更长的PFS相关（2.8 vs 1.7个月，P=0.01）。吉非替尼和Durvalumab治疗EGFR突变晚期NSCLC的I期研究显示^[74]^，PD-L1表达≥20%的患者（n=12）比PD-L1阴性患者（n=24）获得了更好的PFS（15.9 vs 9.1个月）。而根据Lamberti等^[50]^的研究数据，与PD-L1阴性表达的患者相比，PD-L1高表达的EGFR突变NSCLC患者在PFS或OS方面却没有获益。因此，PD-L1表达在预测ICIs对EGFR突变NSCLC患者的疗效的作用中有待进一步研究。

## 4 未来展望

根据以往的回顾性研究和大量临床试验，在驱动基因阴性晚期NSCLC患者中，ICIs显示出令人鼓舞的长期生存优势。然而，EGFR突变晚期NSCLC患者从ICIs治疗中获益有限。免疫单药治疗效果较差，联合靶向治疗会增加AEs风险，ICIs在初治EGFR突变NSCLC患者中并未获得生存优势。免疫+化疗+抗血管生成治疗能否改善EGFR-TKIs耐药晚期NSCLC患者的OS还需要开展更多的临床前和临床研究。此外，未来的研究还应包括联合用药的最佳剂量、顺序和时间安排，以探索EGFR-TKIs和ICIs联合疗法的最佳机制、安全性和疗效。为了最大限度地发挥免疫疗法的作用，研究潜在的预测标志物势在必行。单一的生物标志物，如PD-L1或TMB，可能不足以提供预后价值。整合生物标志物或针对不同患者选择不同的生物标志物将是未来一个新的潜在选择。

**Competing interests**

The authors declare that they have no competing interests.
